# Influential Effects of Intrinsic-Extrinsic Incentive Factors on Management Performance in New Energy Enterprises

**DOI:** 10.3390/ijerph15020292

**Published:** 2018-02-08

**Authors:** Ping Wang, Zhengnan Lu, Jihong Sun

**Affiliations:** 1 School of Management, Jiangsu University, Zhenjiang 212000, China; wangping-01010@163.com (P.W.); lzn@ujs.edu.cn (Z.L.); 2 School of Mental Health, Jining Medical University, Jining 272000, China; 3 National Institute of Education Science, Beijing 100088, China

**Keywords:** new energy enterprises, intrinsic incentive factors, extrinsic incentive factors, work performance

## Abstract

*Background*: New energy has become a key trend for global energy industry development. Talent plays a very critical role in the enhancement of new energy enterprise competitiveness. As a key component of talent, managers have been attracting more and more attention. The increase in job performance relies on, to a certain extent, incentive mechanism. Based on the Two-factor Theory, differences in influences and effects of different incentives on management performance have been checked in this paper from an empirical perspective. *Methods*: This paper selects the middle and low level managers in new energy enterprises as research samples and classifies the managers’ performance into task performance, contextual performance and innovation performance. It uses manager performance questionnaires and intrinsic-extrinsic incentive factor questionnaires to investigate and study the effects and then uses Amos software to analyze the inner link between the intrinsic-extrinsic incentives and job performance. *Results*: Extrinsic incentives affect task performance and innovation performance positively. Intrinsic incentives impose active significant effects on task performance, contextual performance, and innovation performance. The intrinsic incentive plays a more important role than the extrinsic incentive. *Conclusions*: Both the intrinsic-extrinsic incentives affect manager performance positively and the intrinsic incentive plays a more important role than the extrinsic incentive. Several suggestions to management should be given based on these results.

## 1. Introduction

### 1.1. Research Background

In the face of the increasingly severe energy crisis and the trend of environmental deterioration, various countries around the world have been attaching increasing importance on new energies characterized by environmental protection and renewability. The use of new energies not only serves as a solution to energy shortages and represents a pursuit of a low-carbon lifestyle for human beings, but also becomes a breakthrough for sustainable economic development. With the introduction of favorable policies and measures for the new energy industry and the increase in the amount of funds invested in the industry, new energy enterprises have been mushrooming. On the List of Top-500 New Energy Enterprises in the world, Chinese enterprises are dominant in number. At present, the enthusiasm for developing the new energy industry is still running high in various regions in China, and development of the new energy industry has become an important choice for transforming the development mode and adjusting the energy structure. The future development of the new energy industry in China will most likely maintain a high speed. 

The key to continuous and rapid development of new energy enterprises is technological innovation, which serves as the foundation for enhancing their core competitiveness. Only when enterprises have powerful scientific and technological innovation capabilities, can they further enhance their competitiveness and remain invincible in the fierce market competition of the future. The essence of solving issues in technological innovation lies in talent, and the key to competition among enterprises is the competition for talent. How to win the “war for talent” is a tough challenge that new energy enterprises face, which not only means that these enterprises have to find necessary talent in a creative way but also requires them to figure out how to realize the functions of talent and its full potential. To enhance their competitiveness, new energy enterprises need high quality talent as a support. The realization of the effect of intelligence depends on the effect of incentives to a large extent. As a key component of talent, managers have been attracting more and more attention and playing a significant role in achieving organizational goals and strategies. Therefore, how to motivate managers, arouse their enthusiasm and creativity, and improve their job performance has become an important issue in human resources management of new energy enterprises. 

### 1.2. Theoretical Framework and Hypotheses

Scholars and practitioners have always been interested in job performance. At present, the definition of job performance widely accepted in academia is: Job performance is work behavior and outcomes that can be observed and evaluated under a standard formulated by an organization based on its goals, in accordance with the definition proposed by Campbell, Borman and Motowidlo [1,2]. In the traditional structure and dimensional model of job performance that Borman and Motowidlo proposed, job performance consists of task performance and contextual performance. Task performance reflects the behavior of individuals when they are playing their roles and the outcomes; contextual performance reflects their interpersonal and voluntary behavior to facilitate task performance, which helps enhance organizational effectiveness.

Studies on the connotation and structure of job performance always take ordinary employees (rather than managers) as the study subject. Compared to non-management tasks, management is more complicated. Instead of conducting business, managers implement plans, organize, coordinate and oversee employees, instruct departments to complete specific work, and ensure efficiency and orderly implementation of other employees’ work. Therefore, to a large extent, managers achieve goals through others’ work [3]. Does this make the job performance structure of managers different from that of non-managers? 

However, studies on management performance are very limited. Among these limited studies, Coway (1999) divided task performance into two dimensions: technical-administrative task performance and leadership task performance [4]. In another study, he acquired five dimensions of management performance through factor analysis, namely, interpersonal effectiveness, willingness to handle difficult situations, teamwork and personal adjustment, adaptability, and leadership and development [5]. Sun et al. (2002), explored the structure of management performance in Chinese enterprises and the possible factors contained in it [3]. They generalized three dimensions of management performance, namely, task performance, personal quality performance, and relationship performance. As for task performance, its connotation and contained factors are similar to those of Coway’s task management performance. As for personal quality performance and relationship performance, the contents are similar to those of the contextual performance that Borman and Motowidlo proposed. Wen (2005), with middle-level managers in enterprises as the subject, found that job performance could be divided into four structural factors: task performance, interpersonal performance, adaptive performance, and effort performance [6]. In his four-factor model, the definition of task performance is consistent with that of traditional task performance, and the definitions of interpersonal performance and effort performance are similar to those of contextual performance. To a certain extent, the structure and composition of management performance represent a further division of the traditional task performance and contextual performance that Borman and Motowidlo proposed. Although the job description of a manager is different from that of a non-manager, the structure of management performance is almost the same as the traditional structure of job performance. Based on this, this study applies the dimensions of task performance and contextual performance that Borman and Motowidlo have proposed to assess the job performance of managers.

Because global competition is becoming increasingly fierce, the upper hand in enterprise competition is mainly from the ability to be innovative. It is indispensable to be able to cultivate innovative consciousness and develop innovative behavior in employees, especially in managers. Pulakos et al. introduced the dimension of creative problem solving to job performance [7]. Janssen further generalized innovative behavior, and put forward the concept and scale of individual innovative performance, with the scale including innovative willingness, innovative action, innovative achievements, and application of achievements. He also conducted empirical testing, with innovative performance and traditional performance as two dimensions of job performance [8,9]. For this reason, in this study, innovative performance is added to the structure of management performance as a dimension to verify it. 

The study of job performance has become a hot study topic. What aspects will affect the job performance has been one of the major study tasks for researchers. In the late 1950s, American behavioral scientist Fredrick Herzberg and his colleagues discovered after several investigations on employees from different industrial organizations that there were different factors that had influences on satisfaction of employees about their jobs. Those factors directly related to work (such as achievements, job, responsibilities, promotion, etc.) were called “intrinsic factors of work” or “incentive factors”, including work achievements, recognition and appreciation, challenging works, job responsibilities, and opportunities for growth and development that made employees feel satisfied. In addition, they also believed that intrinsic incentive factors played a crucial role in employee motivation.

When feeling the fun, meaning, and autonomy of the job, and getting a sense of accomplishment and self-worth at work, employees will actively behave with a positive attitude. With the effect of intrinsic incentives, individuals will get a string of positive feelings from their work, such as a sense of accomplishment and happiness. Meanwhile, intrinsic incentives pay more attention to how to stimulate employees to work with willingness. Through empirical analysis, Cerasoli (2014) found intrinsic incentives on undergraduates had a significant positive influence on their job performance [10]. Kenneth (2009) pointed out in his research that intrinsic incentives had a significant positive influence on employees’ affective commitments [11]. Ryan believed that intrinsic incentives would bring out the positivity of individuals, who would then set challenging goals for themselves to achieve in their work [12]. Kuvaas (2006), also through empirical analysis, found that intrinsic incentives had a significant positive influence on job performance and affected commitments of employees in financial companies [13]. Based on these previous conclusions, this study proposes:

**Hypothesis** **1.** Intrinsic incentive factors have a significant positive influence on managers’ task performance.

According to the Self-Determination Theory (SDT), self-motivation is generated by individuals who have their own determination and can take control of their own behavior [12]. The main reason for individual behavior is the operation and reflection of one’s own initiative, rather than the outcome of being controlled by the outside world. Intrinsic incentives are an important part of self-motivation [14]. Therefore, when individuals consider a task interesting or meaningful, they will undertake it voluntarily and fulfill it effortlessly. Employees who are given a high degree of intrinsic incentives will not only fulfill their own tasks voluntarily, but also strengthen their communication with colleagues and complete a lot of tasks that don’t fall within their responsibilities but will benefit colleagues, the organization, and the team. So, this study proposes:

**Hypothesis** **2.** Intrinsic incentive factors have a significant positive influence on managers’ contextual performance.

There are many studies on the relationship between intrinsic incentives and individual innovative performance. The most representative is the Componential Model of Creativity by Amabile, which indicates individual creative behavior is influenced by the three factors of creative skills, creative behavior processes, and task motivation [15]. Task motivation is exactly such an intrinsic incentive. When individuals perceive a task as challenging, intriguing, and meaningful, they will put more effort into comprehending it from different perspectives, collect information more widely, and sort it out to bring up more plans to solve problems creatively. The realization of creative behavior can bring a sense of satisfaction and accomplishment to the individual. Intrinsic incentives are the driving force for individual creative behavior. So this study proposes:

**Hypothesis** **3.** Intrinsic incentive factors have a significant positive influence on innovative performance.

Herzberg put forward the concept “extrinsic factors” or “hygiene factors”, which refer to other factors that are not directly related to work, including corporate policies, management measurements, interpersonal relationships, working conditions, and salaries and benefits. These factors may affect employee satisfaction, but won’t stimulate employees directly. Nevertheless, many behavioral scientists believe that factors related to either the workplace or job description may stimulate employees apart from satisfying them. There is a thought prevailing in both theory and management practice that hygiene factors may also stimulate employees in specific contexts [16]. A large number of domestic studies have shown that in general, workplace factors such as salaries, benefits, and interpersonal relationships can stimulate employees in China [17]. With high-skilled employees as the subject, Lv et al. (2010) revealed, through empirical analysis, that salary incentives had a significant influence on the job satisfaction of highly-skilled employees and could strongly explain task performance and contextual performance [18]. Salaries and benefits are intuitive rewards for employees’ work and are closely correlated with their daily lives. Esteem from colleagues and cooperation at work will delight employees and enhance their work efficiency. So, this study proposes Hypothesis 4 and 5.

**Hypothesis** **4.** Extrinsic incentives have a significant positive influence on task performance.

**Hypothesis** **5.** Extrinsic incentives have a significant positive influence on contextual performance.

Scholar Eisenberger (1996), representative of the Behavioral School, proposed the Learned Industriousness Theory. According to the theory, extrinsic incentives can serve as a signal guiding individuals toward a specific creativity-related goal, thus enhancing intrinsic motivation and individual creative behavior [19]. Malik (2015) found that extrinsic incentives had a positive influence on an employee’s creative behavior if the employee had a cognitive style with intrinsic control focus [20]. Hung et al. (2016) found both extrinsic and intrinsic incentives promoted individual creative behavior by positively influencing their intrinsic motivation [21]. So, this study proposes:

**Hypothesis** **6.** Extrinsic incentive factors have a significant positive influence on innovative performance.

## 2. Materials and Methods

### 2.1. Sample Selection and Data Source

This study collected data through questionnaires, with the samples from some new energy enterprises mainly in Shanghai, Jiangsu Province and Zhejiang Province in the Yangtze River delta region. Before the official distribution of the questionnaire, the project team conducted a pre-questionnaire test on three new energy enterprises in Nanjing City of Jiangsu Province, and then altered some items on the questionnaire. Before distributing the questionnaire on a large scale, we contacted 50 enterprises which we randomly selected. With permission from the human resources departments of these enterprises, we obtained email addresses of their middle-level managers and sent the questionnaire to them by email. A total of 500 copies of the questionnaire were distributed. Among the 406 copies returned, 318 were assessed as valid, and the other copies were assessed as invalid due to obvious regularity, lack of answers to many questions, or contradictions. According to statistics, men surveyed accounted for 53.1%, and women accounted for 46.9%; those surveyed with a junior college degree or below accounted for 5.4%, those surveyed with a bachelor’s degree accounted for 67.6%, and those surveyed with a master’s degree or above accounted for 37%; middle-level managers accounted for 68.5%, and primary managers accounted for 31.5%.

### 2.2. Measurement Tools

We designed the initial measurement questionnaire on management performance and intrinsic and extrinsic incentive factors by referring to literature at home and abroad and combining the findings from the open questionnaire of this study. After designing the initial questionnaire, we used an expert survey method and small sample prediction test to revise it. First of all, we asked two professors and three doctoral experts for advice, and made a first revision of the questionnaire. Afterwards, we conducted a small-scale pre-test on MBA students at Jiangsu University. Through preliminary testing and analysis (reliability analysis and exploratory factor analysis), we made a second revision of the questionnaire, and finally obtained a total of 14 measurement questions for the formal questionnaire on management performance and a total of 11 measurement questions for the formal questionnaire on intrinsic and extrinsic incentive factors. All questionnaires were evaluated with the Likert six-point scale to check whether the facts of respondents were consistent with their descriptions, with a rating from 1 (completely inconsistent) to 6 (completely consistent).

The management performance questionnaire included a task performance questionnaire, a contextual performance questionnaire, and an innovation performance questionnaire. Task performance means the behavior reflected during completion of a work task as well as the results, which can be shown by work efficiency, the number and quality of work tasks, and so on. Contextual performance reflects the interpersonal and voluntary behavior to facilitate task performance and completion of organizational work. Innovation performance is reflected by individual innovation actions and refers to the performance of innovations. The task performance questionnaire and the contextual performance questionnaire were formulated based on the job performance model initiated by Borman and Motowidlo (1997) [22]. The innovation performance questionnaire was formulated based on Janssen’s concept and scale of individual innovative performance [8].

As an exploratory factor analysis of the management performance questionnaire found, the *KMO* value is 0.931, and the significance probability of *χ*^2^ in Bartlett’s test of sphericity is 0.000, indicating the data has correlation and is suitable for factor analysis. Three factors have their characteristic values larger than 1, which are 8.348, 1.347, and 1.154, respectively. Together, they explained a 77.501% variation of variance, indicating that the questionnaire items are based on three factors, which represent task performance, contextual performance, and innovation performance respectively, and are consistent with the characteristics of the three dimensions of management performance as described in the hypothesis part of this study.

By referring to the measuring of incentive factors by Price (2001) and Zhang (2011) [23,24], this study measures incentive factors from two dimensions: extrinsic and intrinsic incentives. Extrinsic incentives refer to the incentive in aspects of extrinsic conditions that are not directly related to the work itself. The extrinsic incentive factors include salary and welfare, training and learning, management system, interpersonal relations, working conditions, etc. Intrinsic incentives refer to the incentive produced by the work itself. The intrinsic incentive factors include the following aspects: job autonomy, achievements, position promotion, recognition of performance, display of abilities, and challenge of work. As an exploratory factor analysis of incentive factors found, the *KMO* value is 0.912, and the significance probability of *χ*^2^ in Bartlett’s test of sphericity is 0.000, indicating the data has correlation and is suitable for factor analysis. Two factors have their characteristic values larger than 1, which are 6.062 and 1.337 respectively. Together, they explained a 67.269% variation of variance, indicating that the questionnaire items are based on two factors, which represent extrinsic factors and intrinsic factors.

### 2.3. Reliability and Validity Testing

As found in testing of the scale’s reliability by using the reliability coefficient Cronbach’s *α* (as shown in Table 1), the coefficient *α* of each variable is greater than 0.7—the minimum standard suggested by Chin et al. [25], indicating the scale has high reliability in terms of internal consistency. We checked the validity of the scale through confirmatory factor analysis (CFA). As the findings show, the model has obtained a good effect of fitting (all fitting indexes are within acceptable ranges), and the average variance extracted (*AVE*) of each factor is greater than 0.5—the minimum standard suggested by Bagozzi and Yi (1988) [26], indicating the scale has good convergent validity. 

## 3. Results

### 3.1. Descriptive Statistics of Variables and the Correlation Analysis

We developed descriptive statistics of variables and a correlation analysis, with each variable’s mean value, standard deviation and Pearson correlation coefficient shown in Table 2. The mean value of intrinsic incentive is greater than that of extrinsic incentives, indicating that the organization is more generous to managers in terms of work autonomy, position promotion, and participation in management, but not so generous in terms of welfare and benefits. The mean value of contextual performance being the greatest and the mean value of task performance being the smallest indicate managers’ inertia in their duties and a lack of effective incentives. Meanwhile, innovation performance leaves much to be desired. In terms of correlation of variables, extrinsic incentive has a significant positive correlation with task performance, contextual performance, and innovation performance, and so does intrinsic incentive. The hypotheses thus get proved preliminarily.

### 3.2. Hypothesis Testing Results 

**Figure 1:**
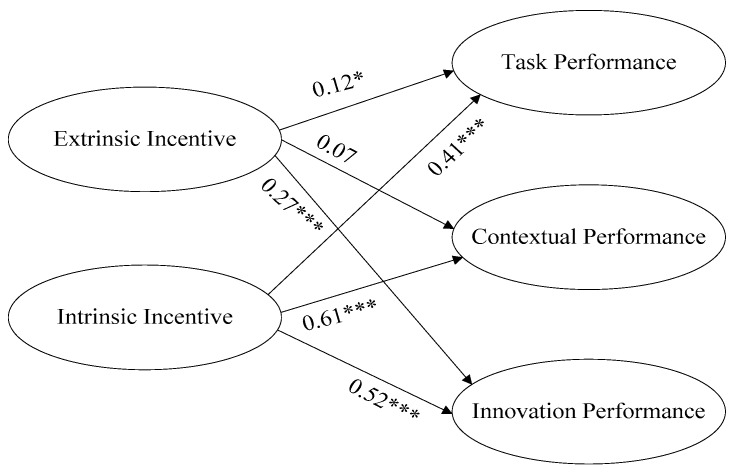
Testing result of influential effect of intrinsic-extrinsic incentive factors on performance. * *p* < 0.1; *** *p* < 0.01.

Through structural equation analysis by using AMOS software, we got the findings as shown in Figure 1. The model’s fitting indexes show: *χ^2^*/*df* = 2.691, *GFI* = 0.859, *NFI* = 0.906, *IFI* = 0.939, *CFI* = 0.939, and *RMSEA* = 0.073. All the indexes are within their acceptable ranges except *GFI* which is slightly lower than its ideal value of 0.9. In a pioneering study for this index, Bollen deemed the value acceptable if it is larger than 0.85 [27]. So the overall collocation degree of the theoretical model is satisfactory.

It can be seen from the Figure 1 that the standardized path coefficient between intrinsic incentive and task performance, contextual performance, and innovation performance is 0.409 (*p* < 0.05), 0.611 (*p* < 0.05) and 0.520 (*p* < 0.05), respectively, indicating intrinsic incentive has a significant positive influence on task performance and innovation performance, thus verifying Hypothesis 1, Hypothesis 2 and Hypothesis 3. 

It can be seen from Figure 1 that the standardized path coefficient between extrinsic incentive and task performance, contextual performance, and innovation performance is 0.116 (*p* < 0.05), 0.074 (*p* > 0.05), and 0.266 (*p* < 0.05), respectively, indicating extrinsic incentive has a significant positive influence on task performance and innovation performance and an insignificant influence on contextual performance. This result supports Hypothesis 4 and Hypothesis 6, and leaves Hypothesis 5 unverified. 

## 4. Discussion

Extrinsic incentives, such as salary and welfare, provided by an organization to its managers will not only meet the basic needs of managers for personal subsistence and family daily life, but also prompt them to better serve the organization, playing a vital role in managers’ duties, innovative behavior, and attitude. First of all, enterprises should provide economic support and health care, set up a scientific management system, and create a comfortable, harmonious workplace. This way, the humanistic care conveyed will help develop a sense of security and belonging and effectively enhance employees’ enthusiasm and dedication, thus propelling work efficiency and boosting department profits. Second, in the process of training and learning, enterprises should develop managers’ competence and hence improve their work efficiency by constantly influencing or changing their cognition, attitudes, and behavior. Also, through training and other welfare incentives, enterprises can strengthen managers’ responsibility and enthusiasm, thus promoting innovation performance.

The main reason Hypothesis 5 is left unverified is: as indicated by the study on contextual performance, what is the most closely related to contextual performance are not external indicators, but intrinsic personalities. Especially after Theories of Personality in working scenarios was advanced, a large number of studies have shown that personality factors including responsibility consciousness and extraversion can significantly improve contextual performance. Personality is the sum of psychological characteristics which have tendency and stability to some extent. Once formed, the personality of an individual tends to settle down and is not likely to change along with extrinsic incentives.

Referring to the existing research, this paper argues that intrinsic incentives mean stimulation brought by work itself to an individual, including methods and strategies to meet moral needs, such as the sense of achievement, being recognized for job performance, personal values being reflected by work, and work autonomy. Empowerment from the organization, as well as support and recognition from superiors, can hugely stimulate managers’ sense of belonging, sense of responsibility and sense of achievement. With these feelings, they will develop more enthusiasm for their work and exhibit more compliant behavior as a member of the organization, which in turn can improve their task efficiency and contextual performance, and have a positive effect on innovation performance through knowledge transfer and sharing among members of the organization [28]. Having appropriate channels for promotion and space for development is one of the main factors affecting employees’ work attitude. Promotion incentives let managers see the prospects for their efforts clearly, and they tend to exhibit valuable and high-quality innovative behavior with more enthusiasm. To achieve their goals, managers will work at full bore to improve their job performance. Challenging work can bring excitement and a sense of calling and urge managers to put more attention to their tasks, which will help stimulate creativity in them [29].

Both intrinsic and extrinsic incentives play a positive role in management performance, but the degrees of effects are not the same, with the effects of the former greater than the latter. Therefore, HR departments should attach more importance to the positive effects brought by intrinsic incentives. According to emotional adaptation theories, extrinsic incentives easily generate emotional psychological adaptation, while intrinsic incentives do not. So, intrinsic incentives are more stable and more durable, and can keep employees being enthusiastic, curious and confident in their work. Compared with intrinsic incentives, the effects of extrinsic incentives are inferior. Gerhart (2009) believed that extrinsic incentives have two functions of classification and stimulation, with the former being more obvious when attracting talent at the very beginning. People cannot experience the intrinsic incentives brought by a specific job when seeking a job. Salaries, rewards and other extrinsic incentives are often what they focus on. Nevertheless, after they get employed, intrinsic incentives play a more manifest role [30].

In terms of the difference between extrinsic incentives and intrinsic incentives, extrinsic incentives lie in material aspects, such as material, money, and awards, to reward employees’ work behavior and outcomes; intrinsic incentives lie in moral aspects, reflecting their competence, sense of accomplishment, self-realization, etc. In extrinsic incentives, organizations pressure employees into their work by means such as hammering away their positions and imposing demanding workloads; in intrinsic incentives, the challenging nature of tasks and the sense of accomplishment give employees strong psychological support and satisfy their needs for success and self-realization, with a sense of responsibility also cultivated in them [31]. So, employees will be more devoted to their own work to achieve better performances.

## 5. Conclusions

By exploring the influences and effects on management performance brought by extrinsic and intrinsic incentives, through empirical verification based on theoretical analysis and logical reasoning, this article now comes to the following conclusion: extrinsic incentives have a positive influence on managers’ task performance and innovation performance, and intrinsic incentives have a significant positive influence on their task performance, contextual performance and innovation performance. In terms of influential effects on management performance, the positive influential effect of intrinsic incentives is greater than that of extrinsic incentives. The conclusion of this study has further confirmed that compared with extrinsic incentives, intrinsic incentives are better at promoting managers’ work behavior and outcomes. 

## Figures and Tables

**Table 1:** The CFA findings of all variables and the coefficient of internal consistency.

	Variable	Cronbach’s *α*	*AVE*	CFA’s Main Indexes for Goodness of Fit
Organizational Incentive Factors	Extrinsic incentives	0.889	0.613	*χ^2^*/*df* = 3.330, *GFI* = 0.897, *CFI* = 0.956, *NFI* = 0.939, *TLI* = 0.956, *RMSEA* = 0.083
	Intrinsic incentives	0.884	0.573	
Management Performance	Task performance	0.909	0.673	*χ^2^*/*df* = 3.976, *GFI* = 0.909, *CFI* = 0.942, *NFI* = 0.942, *TLI* = 0.924, *RMSEA* = 0.067
	Contextual performance	0.911	0.657	
	Innovation performance	0.930	0.764	

CFA: confirmatory factor analysis; *GFI*: goodness of fit index; *CFI*: comparative fit index; *NFI*: normed fit index; *TLI*: Tuck-Lewis index; *RMSEA*: root mean square error of approximation.

**Table 2:** Descriptive statistics of variables and the correlation analysis.

	Mean Value	Standard Deviation	1	2	3	4
1. Task performance	4.65	0.877				
2. Contextual performance	4.95	0.796	0.673 **			
3. Innovation performance	4.69	0.896	0.645 **	0.680 **		
4. Extrinsic incentive	4.39	0.924	0.495 **	0.502 **	0.572 **	
5. Intrinsic incentive	4.50	0.874	0.585 **	0.637 **	0.632 **	0.657 **

Note: ** *p* < 0.05.
